# Comparison of X-ray Radiant Power Absolute Measurement between a Free-Air Ionization Chamber and a Cryogenic Electrical Substitution Radiometer

**DOI:** 10.3390/s23021006

**Published:** 2023-01-15

**Authors:** Fan Li, Yidong Zhao, Peiwei Wang, Kun Tang, Lei Zheng

**Affiliations:** 1 Institute of High Energy Physics, Chinese Academy of Sciences, Beijing 100049, China; 2 National Institute of Metrology, Beijing 100029, China

**Keywords:** comparison, radiant power, free-air ionization chamber, cryogenic radiometer, synchrotron radiation X-ray

## Abstract

Absolute measurement of radiant power in the X-ray region is essential for many applications in astrophysics, spectroscopy, and X-ray diagnostics. Comparison between different measuring methods is an effective way to check their reliability. In the present work, a comparison of X-ray radiant power absolute measurement between a free-air ionization chamber and a cryogenic electrical substitution radiometer was performed at Beijing Synchrotron Radiation Facility. The absolute radiant power obtained by these two methods were mutually compared via a transfer standard detector’s spectral responsivity at a photon energy of 10 keV. The result of the comparison showed that the difference was 0.47%. A conclusion was reached that the free-air ionization chamber and the cryogenic electrical substitution radiometer agreed within the combined relative uncertainty of 3.35%.

## 1. Introduction

Absolute measurement of X-rays’ radiometric quantities with small uncertainties is required in many research fields, e.g., characterization of space instrumentation, X-ray spectrometry, and X-ray diagnostics [1,2,3,4,5,6,7]. Light sources of synchrotron radiation (SR) are powerful tools of fundamental and applied sciences due to their high photon flux and tunability, especially in the X-ray region. Thus, SR-based X-ray radiometry standard is important for related researches and applications [8]. The Physikalisch-Technische Bundesanstalt (PTB) has operated the Metrology Light Source (MLS) as a primary radiation source standard of radiant power from the near infrared radiation up to the soft X-ray region [9,10], and cryogenic electrical substitution radiometers (CESRs) as detector-based radiometry in the region from ultraviolet to hard X-rays [11,12,13]. At the National Institute of Advanced Industrial Science and Technology (AIST), a rare gas ionization chamber in combination with SR has been employed at photon energies up to 3.9 keV [14]. Also, a CESR for SR in soft X-ray region has been developed by AIST [15,16]. At Beijing Synchrotron Radiation Facility (BSRF), the absolute radiant power of SR monochromatized X-ray of 6-20 keV has been obtained by the National Institute of Metrology, China (NIM) using a free-air ionization chamber (FAC) [17,18]. Recently, the Institute of High Energy Physics, Chinese Academy of Sciences (IHEP, CAS) and NIM have been establishing a new SR-based radiometry standard to meet the increasing requirement for calibration service of X-ray detectors [19,20]. A CESR for hard X-rays has been established at beamline 1W1B of BSRF to achieve absolute measurement of X-ray radiant power since 2017.

Comparison is an effective way to investigate the systematic component of the errors of a measurement system. It is also a necessary step to establish a metrology standard. Several comparisons between SR-based radiometry standards have been performed by different national institutes of metrology. A cryogenic radiometer of PTB has been directly compared with a primary radiometric source standard of calculable spectral radiant power, the Berliner Elektronenspeicherring-Gesellschaft fuer Synchrotronstrahlung (BESSY), and the results agreed within the combined relative uncertainties of 2.5 × 10^−3^ [21]. At the SPring-8 extreme ultraviolet free-electron laser, the absolute radiant power obtained by a gas-monitor detector and a CESR were mutually compared and agreed within 2.6% [22].

Although the principles and designs are totally different, both FACs and CESRs measure the radiant power of X-rays absolutely. In the field of metrology, FACs are usually used in a photon energy range from several keV to hundreds of keV, and CESRs are used in the visible, ultraviolet and X-ray spectral regions, in which the photon energies are up to 60 keV. A comparison between radiant power measured using a FAC and a CESR in the X-ray region can help investigate the measurement errors and establish mutual compatibility.

In the present work, a comparison between absolute radiant power measured using the FAC of NIM and the CESR of IHEP was performed at BSRF in the X-ray region to check their reliabilities, and a preliminary result was reported.

## 2. Method and Instrumentation

An indirect comparison was carried out between the FAC and the CESR because they were performed at beamline 4W1A and 1W1B, respectively. Thus, a photodiode was employed to be the transfer standard detector and its spectral responsivity was calibrated against the FAC and the CESR at the photon energy of 10 keV, respectively. Because photodiodes have proven to be highly stable and were used as the transfer detector for other radiometric international comparisons, the spectral responsivity of the photodiode was used to represent the radiant power measured by the FAC and the CESR, since a direct comparison was unavailable.

The main criterion used to assess the result of the comparison is as follows:

(1) $$\left|s_{1}-s_{2}\right|\leq \sqrt{U_{1}^{2}+U_{2}^{2}}.$$

Here, *s*_1_ denotes the measured value by the first method, namely the spectral responsivity of the photodiode calibrated against the FAC; *s*_2_ denotes the measured value by the second method, namely the spectral responsivity of the photodiode calibrated against the CESR; *U*_1_ denotes the uncertainty of *s*_1_; and *U*_2_ denotes the uncertainty of *s*_2_. When Equation (1) is fulfilled, the two methods agree with each other. 

In the comparison, the optic characteristics of the beamlines were investigated to estimate the uncertainties caused by light sources. The FAC and the CESR were closely studied and their uncertainties were estimated. The photodiode’s dependence of the spectral responsivity on the radiant power and spatial homogeneity of the spectral responsivity were measured to estimate the corresponding uncertainties.

### 2.1. Monochromatized Synchrotron Radiation Sources

BSRF operates more than 10 beamlines at the electron storage ring Beijing Electron Positron Collider II (BEPC II) to provide SR in the range from vacuum ultraviolet to hard X-rays. The FAC and the CESR of this comparison are performed at beamline 4W1A and 1W1B, respectively. 

At 4W1A, a 1.8 T wiggler beamline, a photon energy region of 6–22 keV is available [23,24]. A silicon channel-cut double crystal monochromator (DCM) and an InSb crystal are used to achieve a high spectral resolution and high spectral purity. Additionally, the photon energy scale has been calibrated to better than 10 eV by using the energy of K absorption edges of Cu, Ge, Se, Sr, Zr, and Mo. The beam size is about 4 mm in diameter, which is smaller than the entrance of the FAC. SR-based radiometry and calibration has been studied here using the FAC for several years. During the experiment at 4W1A the storage ring was operated in top-up mode with electron energy of 2.5 GeV and current of 500 mA.

At 1W1B, a 1.28 T wiggler beamline, a photon energy region of 4–25 keV is available [25]. A plane mirror coated with Ni, a silicon DCM and a toroidal mirror are used to combine a high spectral resolution and low higher-order contributions in the region of interest. The photon energy scale has been calibrated to better than 10 eV by using the well-known energies of the absorption edges of appropriate elements, such as Ni, Cu, Ge, Y, Zr, Mo, etc. The beam size is about 2 mm × 3 mm at 10 keV, which is smaller than the entrance of the CESR. The CESR for hard X-rays has been operated here since 2017. During the experiment at 1W1B the storage ring was operated in decay mode, in which the electron energy was 2.2 GeV and current was decreasing from 600 mA to 450 mA with a lifetime of about 3 h. The photon beam intensity at 10 keV varied from 4 μW to 90 μW due to the storage ring current. An ionization chamber was employed to monitor the fluxes of the SR X-rays.

The uncertainties introduced by light sources are mainly caused by the error of energy scale and harmonic. According to the theoretical model of the photodiode’s spectral responsivity [17,26], a −eV photon energy error causes an uncertainty of 0.02% around 10 keV, and the spectral impurity causes an uncertainty below 0.40%. In total the uncertainties caused by the light sources are estimated to be 0.40%.

### 2.2. Free-Air Ionization Chamber

**Figure 1:**
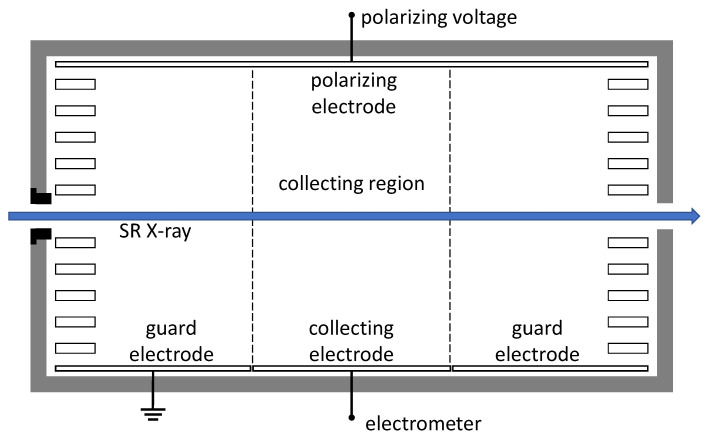
Simplified schematic diagram of a free-air ionization chamber. Photons enter and interact with the air in the chamber to produce electron-ion pairs. Charge in the collecting region is collected and measured as an ionization current.

The principle design of the parallel plate FAC used in this comparison experiment is shown in the sketch of Figure 1 [17]. It consists mainly of an entrance diaphragm with aperture diameter of 5 mm and a pair of planar electrodes separated by a distance of 60.5 mm. One of the electrodes is set to ground potential while a certain polarizing potential is applied to the other to produce an electric field between them, and the latter electrode is referred to as the polarizing electrode. Because of the electric field the electron-ion pairs generated by interactions of photons with air are separated. The polarizing potential was chosen to be 2400 V according to a measured plateau curve. The electrode of ground potential is separated to collecting electrode and guard electrode. The collecting electrode has a length of 40.5 mm in the beam direction, and is connected to a sensitive current measurement system. The collecting electrode and the electric field lines define a so-called collecting region, in which charge is collected and the current carried by ions is measured. A series of resistors is mounted on all four sides in the FAC, shown as small rectangles in Figure 1. Each resistor is set at the exactly desired potential of the electric field at the mid-height of that resistor in order to make the electric field in FAC as uniform as possible.

With X-ray mass energy-absorption coefficients of air, FACs can be used to measure the energy flux absolutely [13]. Hence absolute radiant power of monochromatized SR beams could be obtained by averaging the energy over time. For a given photon energy *E* the radiant power *P(E)* is obtained from the measured ionization current *I_ion_* in a FAC by [17]:

(2) $$P\left(E\right)=\frac{I_{ion}\left(W_{air}/e\right)}{\frac{\mu_{en}}{\rho }\left(E\right)\rho d}\prod_{i}k_{i}.$$

Here, *W_air_* denotes the mean energy for electron-ion pair creation in air; *e* denotes the elementary charge; *μ_en_/ρ* denotes the X-ray mass energy-absorption coefficients of air; *ρ* denotes the density of air in the FAC; *d* denotes the length of collecting region; and *k_i_* denotes the correction factors, which are correction factor *k_s_* for recombination, *k_h_* for humidity, *k_d_* for electric field distortion, *k_e_* for electron loss, *k_pol_* for polarity, *k_sc_* for scattering photons, and *k_fl_* for fuorescence photons. 

A complete uncertainty budget of a measurement at 10 keV is given in Table 1, where *X_i_* denotes the input quantity, *x_i_* denotes the estimation of input quantity *X_i_*, *u(x_i_)* denotes the standard uncertainty of the input estimate *x_i_*, and *u_c_* denotes the combined standard uncertainty of the output quantity radiant power. Obviously, the uncertainty of *µ_en_/ρ* of 0.8% is the dominant contribution to the uncertainty of radiant power.

### 2.3. Cryogenic Electrical Substitution Radiometer

**Figure 2:**
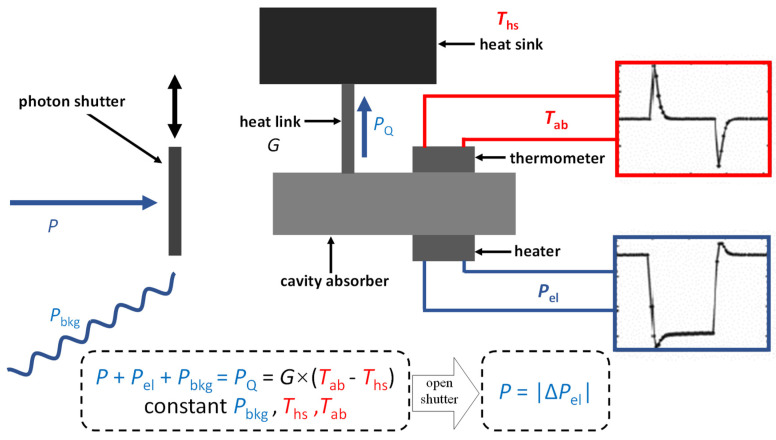
Operating principle of the CESR. Red symbols indicate status of temperature, and blue symbols indicate the flow of power.

The core piece of the CESR is its cavity absorber, which is thermally linked to a heatsink with fixed temperature. Both the cavity absorber and heatsink are equipped with a thermometer and a heater to allow for the temperature monitoring and the supply of electrical heating power. The absorber-heatsink system is cooled down below 3 K by a cool head (Sumitomo Heavy Industries, Ltd. RDK-408D2) and a compressor (Sumitomo (SHI) Cryogenics of America, Inc. HC-4E1). The temperature of the heatsink is kept at 3 K by an active control. The cavity absorber is kept at a higher temperature by another active control. When incident SR is provided, the electrical heating power required to keep the cavity absorber at a constant temperature undergoes a reduction equivalent to the incident radiant power, so that the radiant power is obtained through the measurement of the electrical heating power (see Figure 2). The incident radiant power *P* is obtained by the following:

(3) $$\eta P=G\cdot \Delta T-P_{el}-P_{bkg}=G\cdot \left(T_{ab}-T_{hs}\right)-\frac{V_{htr}\cdot V_{std}}{R_{std}}-P_{bkg}.$$

Here, *η* denotes the absorptance of the cavity; *G* denotes the thermal conductance of the heat link; Δ*T* denotes the difference between temperature of absorber *T_ab_* and heatsink *T_hs_*; *P_el_* denotes the power of the heater, which is derived from its voltage *V_htr_* and current, and the current is calculated from resistance *R_std_* of a standard resistor and the voltage *V_std_* of the resistor; and *P_bkg_* denotes the power of background radiation.

**Figure 3:**
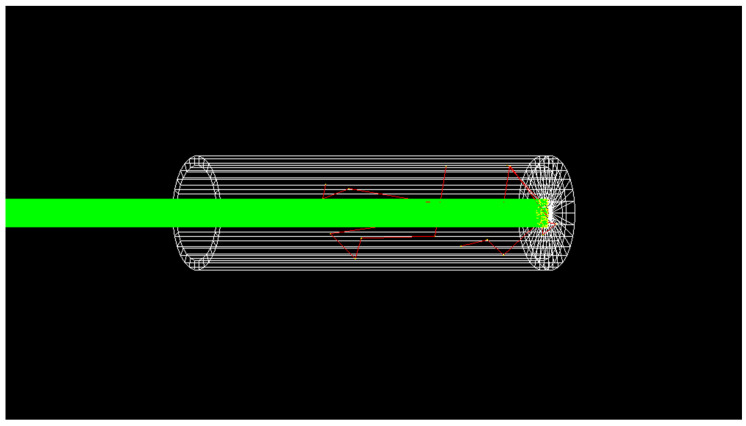
Model of the cavity absorber using Geant4. The absorber is a cylinder with a bottom (white wireframe). Incident photons are mainly absorbed by the cylindrical copper bottom. Scattered photons, fluorescence photons, and electrons produced by interactions between X-rays and copper are reabsorbed by the cylindrical copper shell. The green lines represent trajectories of the photons and the red lines represent trajectories of the electrons.

The cavity absorber of the CESR is made of copper, which provides excellent thermal conductivity at cryogenic temperature and a moderate heat capacity, which ensures a short response time suitable for the measurement of monochromatized SR. The geometry of the cavity was designed to have the most absorptance, and the Monte Carlo simulation code Geant4 [27] was applied to calculate its absorptance for photon energies of up to 60 keV in which the possible loss mechanisms due to reflection, transmission, and scattering, as well as the emission of photoelectrons and fluorescence radiation were investigated (see Figure 3). The cavity absorber has a diameter of 14 mm and its bottom thickness is 1.5 mm. The length of the cavity absorber measures 21.5 mm and the thickness of the cylindrical shell of cavity absorber is 1 mm. The simulation shows that the absorptance of the cavity absorber is about 1 × 10^−5^ different from unity at 10 keV.

**Figure 4:**
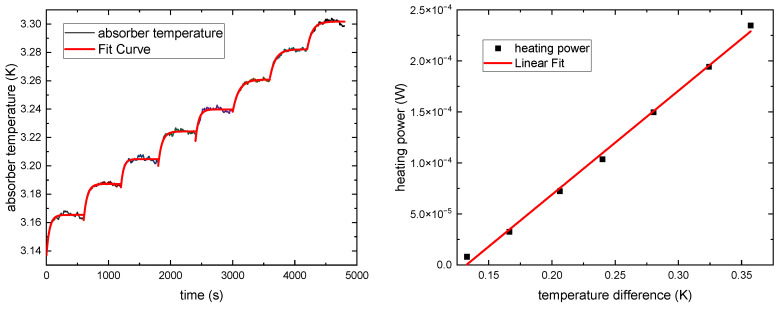
Experimental data for determining time constant *τ* of the cavity absorber (**left**) and correlation between heating power and cavity absorber temperature to calculate the thermal conductance *G* of the heat link (**right**).

The thermal properties of the CESR, including the thermal time constant *τ* of the cavity absorber and the thermal conductance *G* of the heat link were determined experimentally. The thermal time constant *τ* corresponds to the time that the cavity absorber requires to diminish the temperature difference Δ*T* by a factor of 1/e without applying the dynamic substitution mode. It was measured by applying a certain electrical heating power to the cavity absorber and monitoring the temperature, then fitting the time-temperature function. The time constant was measured at different absorber temperatures and the results are shown in Figure 4. The cavity absorber was set at 3.24 K and had a thermal time constant of about 50 seconds, thus the corresponding time taken to perform a measurement was less than 10 minutes, and the cavity absorber heater’s electrical power was higher than incident X-ray power, so that an active control to keep the cavity absorber’s temperature constant could be achieved.

With the cavity absorber at temperature *T*, the thermal conductance *G(T)* of the heat link is given by the ratio between the rise in applied heating power Δ*P* of the cavity absorber’s heater and the temperature rise Δ*T* of the cavity absorber:

(4) G(T)=ΔP/ΔT.

In Figure 4, the correlation between the respective heating power and the cavity absorber temperature is shown, from which the thermal conductance can be determined according to Equation (4). When the cavity absorber was at 3.24 K, the thermal conductance was determined to be 1.02 × 10^−3^ W/K. The thermal conductance helped to set other parameters of CESR and was used to evaluate the uncertainty of the CESR.

While the cavity absorber and heatsink were performed in vacuum, a 20-µm thick beryllium window was installed for vacuum isolation at the entrance of the CESR which had a diameter of 8 mm. Besides, three more beryllium windows were installed at each layer of radiometer (which had different temperatures) to suppress the radiation heating power from the entrance. The thicknesses of all the beryllium windows were measured accurately before the installation.

A complete uncertainty budget of a measurement using the CESR at 10 keV is given in Table 2 as an example, where *X_i_* is the ith input quantity, *µ_A_* and *µ_B_* are the type A and type B standard uncertainty of the input *X_i_*, *C_i_* is the sensitivity coefficient, and *u_c_* is the combined standard uncertainty of the output quantity radiant power. In the experiment, the average radiant power of incident X-ray was about 4 × 10^−5^ W, so the relative uncertainty of the CESR was estimated to be 1.10%. 

### 2.4. Silicon Photodiode

A transfer standard detector is needed to perform an indirect comparison. Silicon photodiodes are easy-to-operate detectors and have stable performances which make them appropriate to be transfer standards. For the present experiment, a Hamamatsu type S3590-08 photodiode was used as the transfer standard detector. Before the comparison, the photodiode’s dependence of the spectral responsivity on the radiant power and spatial homogeneity of the spectral responsivity were investigated.

**Figure 5:**
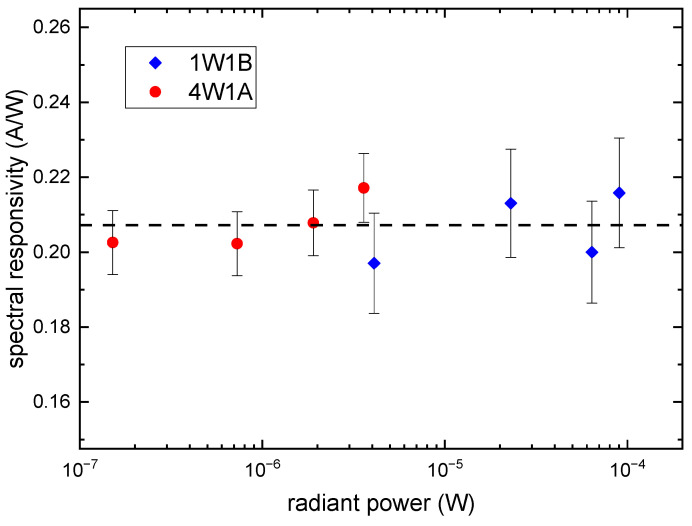
Dependence of the spectral responsivity on the radiant power for the semiconductor photodiode. The dashed line indicates the average spectral responsivity of photodiode.

The FAC and the CESR were performed at 4W1A and 1W1B, respectively, and the beam intensities of 4W1A and 1W1B were quite different. Although both beam power ranges of 4W1A and 1W1B were covered by the FAC and the CESR, an indirect comparison was conducted because of the limitation of the experimental stations. This required the spectral responsivity of the photodiode to be independent to the radiant power in the region of interest. The dependence of the spectral responsivity on the radiant power for the photodiode was studied. The photodiode was calibrated at beamline 4W1A with a beam power range from 1 × 10^−7^ W to 4 × 10^−6^ W, and at beamline 1W1B with a beam power range from 4 × 10^−6^ W to 1 × 10^−4^ W. The result is shown in Figure 5. The uncertainty caused by the imperfect linearity of photodiode’s responsivity was estimated to be 2.39%. The influence of SR energy on the linearity is negligible for the photon energy error of light source is below 10 eV.

**Figure 6:**
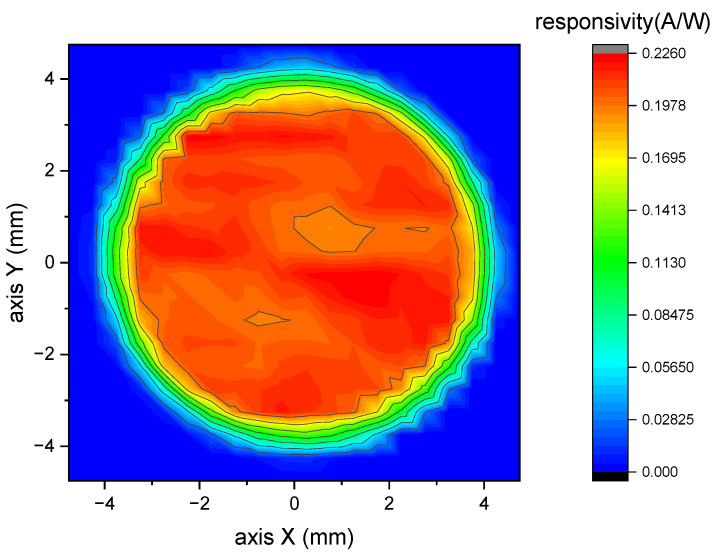
Spatial distribution of the spectral responsivity for the photodiode.

SR beams’ cross-sectional areas were confined to be smaller than the entrances of the FAC and the CESR to make sure the incident X-ray fluxes being measured totally. Different positions of the photodiode were exposed to X-rays in different experiments and this gave rise to the requirement for the spatial homogeneity of the responsivity. Thus, the spatial homogeneity of the responsivity was measured to ensure the reliability of the indirect comparison. The result is shown in Figure 6. The uncertainty caused by the imperfect spatial homogeneity of photodiode’s responsivity was estimated to be 1.11%.

## 3. Experiment

**Figure 7:**
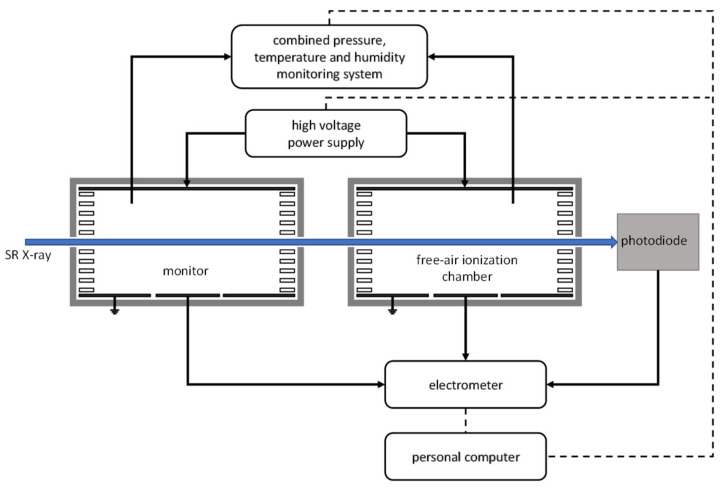
Schematics of the absolute radiant power measurement and calibration system using the FAC.

**Figure 8:**
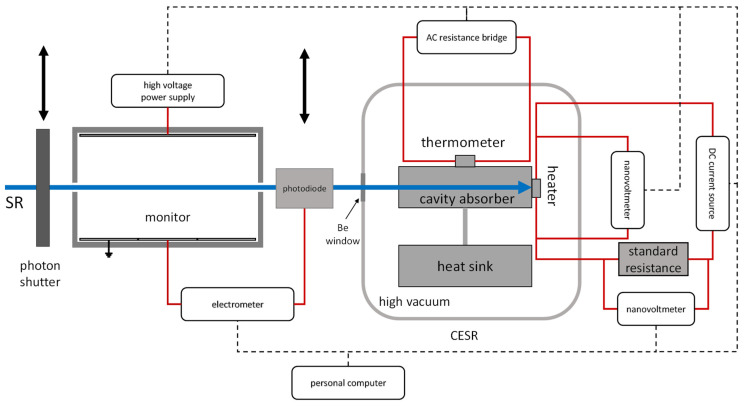
Schematics of the absolute radiant power measurement and calibration system using the CESR. Red solid lines indicate measurement signal and supply cables, and dashed lines indicate control signal cables.

There were two experiments in the indirect comparison, in which the transfer standard detector was calibrated against the FAC and the CESR sequentially. The set-ups are schematically shown in Figure 7 and Figure 8. 

In the calibration against the FAC at beamline 4W1A, the responsivity of photodiode *s*_1_ in units of A/W, can be written as follows:

(5) $$s_{1}=\frac{I_{dio}}{P\cdot \mu_{Be}\cdot \mu_{air}}$$

Here, *I_dio_* denotes the measured ionization current of the photodiode, in units of A; *P* denotes the measured radiant power by the FAC, in units of W; *μ_Be_* denotes the transmission rate of Be window of the photodiode, in dimensionless units; *μ_air_* denotes the transmission rate of air between the reference planes of the FAC and the photodiode, in dimensionless units. The *μ_Be_* was measured and *μ_air_* was calculated from the mass attenuation coefficient *μ/ρ* of air, the density of air, and the distance between the reference planes of FAC and the photodiode.

The currents of FAC and the photodiode were measured simultaneously to avoid the influence of decreasing flux. This could be executed because the FAC worked in transmission mode. A monitor was used to measure the FAC’s correction factors, such as *k_s_* for recombination.

In the calibration against the CESR at beamline 1W1B, the responsivity of photodiode *s*_2_ can be written as follows:

(6) $$s_{2}=\frac{I_{dio}}{P}\frac{M_{P}}{M_{I}}\cdot \mu_{Be}\cdot \mu_{air}$$

Here, *I_dio_* denotes the measured current of the photodiode, *P* denotes the measured radiant power by the CESR, *M_P_* denotes the monitored beam intensity while the CESR was measuring, and *M_I_* denotes the monitored beam intensity while the photodiode was measuring. *M_P_* and *M_I_* are dimensionless.

Neither of the CESR nor the photodiode could work in transmission mode, so the beam intensity was measured sequentially using the CESR and the photodiode. An electric motor was applied to make the photodiode enter and exit the photon beam. Because the photon beam intensity decreased during the experiment and the detectors measured at different time, a correction was made on the photodiode current using the signal measured with a monitor, which was a 50%Ar_2_ and 50%N_2_ ionization chamber. When the CESR or photodiode was measuring, the monitor detected the beam intensity as *M_P_* or *M_I_*, respectively, and the photodiode current was corrected using *M_P_/M_I_*.

## 4. Results and Discussion

The average of measured responsivities of the photodiode was adopted as the final result of the calibration. The result of calibration against the FAC is 0.20747 A/W with an uncertainty of 1.11%, and the result of calibration against the CESR is 0.20649 A/W with an uncertainty of 1.74%. The uncertainty budgets of the photodiode responsivity measurements using the FAC and the CESR at 10 keV are given in Table 3.

The uncertainty budget of the comparison experiment between the FAC and the CESR at 10 keV is given in Table 4. All input quantities are independent and the usual method of “root-sum-of-squares” was used for combining standard deviations. The difference between results of the FAC and the CESR is 0.47%, much smaller than the uncertainty of the comparison experiment, also smaller than the uncertainties of the FAC and the CESR. Thus, Equation (1) is fulfilled and the absolute radiant power obtained by the FAC and the CESR agree with each other within the uncertainty of 3.35%.

In the present work, the systematic uncertainties contribute considerably to the uncertainty of the FAC and the statistical uncertainties contribute dominantly to the uncertainty of the CESR. The main contributions to the uncertainty of radiant power measured by the FAC are the uncertainties of *µ_en_/ρ* and *W_air_/e*. It is a difficult task to improve the accuracy of constants such as *µ_en_/ρ* and *W_air_/e*, as well as the accuracy of the FAC. For the CESR, the fluctuation of the background radiation is the dominant contribution to the uncertainty, because the background radiation could not be suppressed due to the constraints of the experimental station’s space. With a new SR light source, the High Energy Photon Source (HEPS) under construction [28], the CESR will be improved significantly in the future.

## 5. Conclusions

A comparison of the absolute radiant power measured using the FAC and the CESR was performed at the photon energy of 10 keV. The light sources, the FAC, the CESR and the transfer standard detector were closely studied and their uncertainties were estimated. The transfer standard detector was calibrated against the FAC and the CESR at beamline 4W1A and 1W1B, respectively. A preliminary result was reported as that the difference between the FAC and the CESR was 0.47%. It is concluded that the radiant power measured by the FAC and the CESR agree within 0.47% with the uncertainty of 3.35%. In the future, the CESR will be improved at a metrology customized beamline of the forthcoming 4th generation SR source HEPS. Additionally, the photon energy region of the comparison will be extended on the basis of the new light source.

## Figures and Tables

**Table 1:** Contributions to the relative standard uncertainty of radiant power *P* (*k* = 1) for measurement with the FAC at photon energy of 10 keV. The term *k* is the coverage factor for the expanded uncertainty.

*X_i_*	*x_i_*	*u*(*x_i_*) (%)
*I*	1.51 × 10^−9^ A	0.22
*W_air_*	33.97 eV	0.35
*µ_en_/ρ*	4.60 m^2^kg^-1^	0.80
*ρ*	1.19 kgm^−3^	0.07
*d*	4.05 × 10^−2^ m	0.01
*k_s_*	1.0015	0.02
*k_h_*	0.9980	0.03
*k_d_*	1.0000	0.01
*k_e_*	1.0000	0.05
*k_pol_*	1.0000	0.05
*k_sc_k_fl_*	0.9962	0.10
result	Value	*u_c_* (%)
radiant power	2.29 × 10^−6^ W	0.92

**Table 2:** Contributions to the standard uncertainty of radiant power P (*k* = 1) for measurement with the CESR at photon energy of 10 keV.

*X_i_*	*μ_A_*	*μ_B_*	*C_i_*
Δ*T*	3.96 × 10^−5^ K	-	0.00102 W/K
*V_std_*	-	8.7 × 10^−6^ V	−1.32 × 10^−4^ V/Ω
*V_htr_*	-	8.7 × 10^−6^ V	−8.01 × 10^−5^ V/Ω
*R_std_*	3.69 × 10^−4^ Ω	5.9 × 10^−2^ Ω	1.07 × 10^−8^ V^2^/Ω^2^
*P_bkg_*	-	4.4 × 10^−7^ W	−1
*H*	-	0.001	4 × 10^−5^ W
result		combined standard uncertainty u_c_	
Radiant power		4.39 × 10^−7^ W	

**Table 3:** Uncertainties of calibrations of the photodiode spectral responsivity (*k* = 1) against the FAC and the CESR.

Sources	Calibration against FAC		Calibration against CESR	
	Value	Uncertainty (%)	Value	Uncertainty (%)
*I_dio_*	6.13 × 10^−5^ A	0.13	9.77 × 10^−5^ A	0.12
*P*	3.59 × 10^−6^ W	0.92	4.09 × 10^−6^ W	1.10
*M_P_*/*M_I_*	-	-	-	0.10
*μ_Be_*	0.98963	0.01	0.98963	0.01
*μ_air_*	0.83149	0.51	0.87347	0.41
Light source	-	0.40	-	0.40
Type A	-	0.01	-	1.21
Combined	0.20747 A/W	1.11	0.20649 A/W	1.74

**Table 4:** Uncertainty of the comparison experiment (*k* = 1).

Sources	Value	Uncertainty (%)
*s*_1_	0.20747 A/W	1.11
*s*_2_	0.20649 A/W	1.74
Linearity of photodiode	-	2.39
Spatial homogeneity of photodiode	-	1.11
Combined	-	3.35

## Data Availability

Not applicable.
